# Predictive value of derived neutrophil-to-lymphocyte ratio for immune checkpoint inhibitor outcomes in advanced gastric cancer

**DOI:** 10.1097/MD.0000000000046503

**Published:** 2025-12-12

**Authors:** Yu Cao, Hangjun Gong, Gang Han, Yun Zhang, Yanyan Zhang, Xu Zhang

**Affiliations:** a Department of Gastrointestinal Surgery, East Hospital of Shuguang Hospital, Shanghai, China.

**Keywords:** advanced gastric cancer, derived neutrophil-to-lymphocyte ratio, immune checkpoint inhibitors, prognostic biomarker, progression-free survival

## Abstract

The derived neutrophil-to-lymphocyte ratio (dNLR) has been suggested as a potential prognostic biomarker for patients undergoing immune checkpoint inhibitor (ICI) therapy, particularly in advanced gastric cancer (AGC). This study aims to evaluate the prognostic value of pretreatment dNLR in AGC patients receiving ICIs. In this retrospective study, 168 AGC patients treated with ICIs at our hospital from January 2015 to December 2019 were included. Patients were grouped based on baseline dNLR levels: high (dNLR ≥ 3) and low (dNLR < 3). Treatment response was evaluated using the response evaluation criteria in solid tumors version 1.1, and progression-free survival (PFS) and overall survival (OS) were assessed using Kaplan–Meier survival analysis. Statistical analyses included Cox proportional hazards regression and log-rank tests to determine the impact of dNLR on survival outcomes. A total of 168 patients with AGC were analyzed. Baseline characteristics were generally balanced between groups, with 22.6% of patients classified as high dNLR (≥3). Treatment response did not significantly differ according to dNLR status: objective response rate was 26.2% in the low dNLR group versus 23.7% in the high dNLR group, and disease control rate was 49.2% versus 44.8%, respectively. In contrast, survival outcomes showed significant differences. Patients with low dNLR had a markedly longer median OS (23.5 vs 15.6 months; hazard ratio = 1.865, 95% confidence interval: 1.216–3.156, *P* < .001) and median PFS (15.6 vs 8.5 months; hazard ratio = 1.916, 95% confidence interval: 1.213–3.028, *P* < .001) compared with those with high dNLR. Multivariate Cox regression adjusting for age, Eastern Cooperative Oncology Group score, metastatic burden, and treatment modality confirmed that high dNLR independently predicted poorer PFS and OS. Pretreatment dNLR is a valuable prognostic biomarker for PFS and OS in AGC patients receiving ICI therapy. Patients with lower dNLR levels may achieve more favorable outcomes, underscoring the potential of dNLR in guiding personalized treatment strategies for AGC.

## 1. Introduction

Advanced gastric cancer (AGC) remains one of the leading causes of cancer-related mortality globally, accounting for significant morbidity due to its aggressive nature and poor survival outcomes. As the disease frequently presents at advanced stages, traditional treatment modalities – such as chemotherapy, radiotherapy, and targeted therapy – have shown limited efficacy in achieving long-term remission or substantial improvement in survival rates.^[1–3]^ In recent years, immune checkpoint inhibitors (ICIs) have emerged as a promising treatment option for patients with various advanced malignancies, including AGC. ICIs, such as programmed death-1 and programmed death-ligand 1 (PD-L1) inhibitors, function by blocking immune checkpoint pathways that typically downregulate immune responses, thereby reactivating T cells against tumor cells.^[4,5]^ However, despite the advancements in immunotherapy, the response to ICIs in AGC is variable, necessitating the identification of reliable biomarkers that can predict therapeutic outcomes and guide treatment decisions effectively.

Biomarkers for response prediction in immunotherapy have been extensively researched, particularly in the context of ICIs, to aid clinicians in patient stratification and prognostic evaluation. Among the markers explored, PD-L1 expression on tumor cells, microsatellite instability status, and tumor mutational burden have garnered attention; however, these biomarkers often require invasive tissue biopsies and may not consistently correlate with clinical outcomes in AGC.^[6,7]^ Consequently, there is an increasing demand for simple, noninvasive, and reproducible biomarkers that accurately reflect the immune-inflammatory environment and provide prognostic insights. In this regard, hematological biomarkers derived from routine blood tests, such as the neutrophil-to-lymphocyte ratio (NLR), have emerged as potential indicators due to their simplicity, accessibility, and potential prognostic value. The derived neutrophil-to-lymphocyte ratio (dNLR) has shown promise as a systemic inflammatory marker that may correlate with the immune status of patients and predict response to ICIs. The dNLR, calculated by dividing the absolute neutrophil count by the sum of absolute lymphocyte count and monocyte count, is a modified version of the traditional NLR. Elevated dNLR values have been associated with a pro-inflammatory state and an immunosuppressive tumor microenvironment, potentially indicating a reduced response to immunotherapy. Furthermore, studies across various cancers have demonstrated that dNLR may serve as an independent prognostic factor, reflecting the systemic inflammatory response that is often linked to disease progression and poorer survival outcomes.^[8,9]^ However, systematic validation of dNLR in the specific context of gastric cancer patients treated with ICIs remains limited. Previous studies have mainly focused on mixed tumor populations or non-ICI treatment settings, used heterogeneous cutoff values, and rarely incorporated multivariate analyses to confirm its independent prognostic value. Moreover, few investigations have evaluated its predictive role for both progression-free survival (PFS) and overall survival (OS) in real-world AGC cohorts, particularly within Asian populations.

Given these gaps, the present study aims to clarify the prognostic significance of pretreatment dNLR in AGC patients receiving ICIs and to determine whether dNLR can independently predict survival outcomes after adjusting for key clinical variables. By establishing the relationship between dNLR and therapeutic outcomes, this study seeks to provide further evidence for the utility of this easily obtainable biomarker and to contribute to optimizing immunotherapy strategies for AGC.

## 2. Materials and methods

### 2.1. Study design

This study was approved by the Ethics Committee of East Hospital of Shuguang Hospital.This retrospective evaluation was conducted at our hospital to assess the dNLR as a predictive biomarker for the prognosis of ICI therapy in patients with AGC. The study covered the period from January 2015 to December 2019, during which 168 patients with AGC who received ICI treatment at our hospital were included in the analysis. Research methodology, study objectives, and protocols were rigorously developed following the Strengthening the Reporting of Observational Studies in Epidemiology guidelines to ensure the robustness and transparency of the observational data.^[10]^ Informed consent was obtained from all subjects and/or their legal guardian(s). The study’s methodology and protocols were reviewed and approved by the hospital’s ethics committee. All procedures adhered to the relevant guidelines and the ethical principles outlined in the Declaration of Helsinki. Data was handled confidentially, with personal identifiers removed prior to analysis to ensure participant privacy.

### 2.2. Inclusion and exclusion criteria

#### 2.2.1. Inclusion criteria

1) Diagnosis of AGC: patients diagnosed with histologically confirmed AGC (stage III or IV).
2) Treatment with ICIs: patients who have received at least 1 cycle of ICI therapy.
3) Complete clinical data availability: patients with comprehensive baseline clinical and laboratory data, including neutrophil and lymphocyte counts, to facilitate accurate calculation of the dNLR.
4) Follow-up data: patients with documented follow-up information to assess response to ICI therapy and OS outcomes.

#### 2.2.2. Exclusion criteria

1) Concurrent malignancies: patients with a history of or concurrent diagnosis of other malignancies, except for nonmelanoma skin cancer or localized prostate cancer, to avoid confounding survival outcomes.
2) Prior ICI therapy: patients who received ICIs as part of a prior line of therapy before the study period, to ensure the uniformity of treatment exposure.
3) Autoimmune diseases or immunosuppressive therapy: patients with preexisting autoimmune diseases requiring active immunosuppressive therapy, as this may influence immune response and skew the efficacy of ICI treatment.
4) Severe infectious or inflammatory conditions: patients with active severe infections or inflammatory conditions at baseline, which could significantly affect neutrophil or lymphocyte counts, potentially confounding the dNLR measurements.

### 2.3. Treatment protocols

The treatment regimens for patients included a range of ICIs therapies and combinations tailored to individual needs: ICIs monotherapy, ICIs combined with chemotherapy, ICIs with antiangiogenic therapy, and a combination of all 3. ICIs administered intravenously included toripalimab (240 mg), sintilimab (200 mg), nivolumab (3 mg/kg), and pembrolizumab (2 mg/kg), with CT/MRI evaluations conducted every 3 cycles. Chemotherapy protocols varied: the XELOX regimen included capecitabine (1000 mg/m²) orally twice daily for 14 days followed by a 7-day rest, with oxaliplatin (130 mg/m²) on day 1; the SOX regimen utilized S-1 (40–60 mg) orally twice daily for 14 days with a 7-day rest, combined with oxaliplatin (130 mg/m²) on day 1; and the DCF regimen involved intravenous fluorouracil (750 mg/[m²·day]), with docetaxel (75 mg/m²) and cisplatin (75 mg/m²) on day 1. Antiangiogenic agents included bevacizumab (5 mg/kg biweekly or 7.5 mg/kg triweekly) and oral apatinib (850 mg daily after meals). Each treatment approach was customized based on disease severity, patient health, and personal preferences to optimize therapeutic outcomes.

### 2.4. Outcome measures

The dNLR was calculated as the absolute neutrophil count divided by the sum of the absolute lymphocyte count and the monocyte count (dNLR = N/[L + M]). This operational definition has been reported in prior studies and is mathematically equivalent to the more commonly cited formula, dNLR = N/(WBC – N), given that WBC = N + L + M. We adopted this definition to ensure clarity and consistency with our available laboratory data, which provided neutrophil, lymphocyte, and monocyte counts directly. Throughout this study, this formula was consistently applied in all analyses. Based on baseline dNLR levels, patients were classified into 2 groups using a threshold of dNLR = 3. This cutoff value was selected with reference to prior international clinical studies, where dNLR ≥ 3 has been consistently applied as a clinically relevant prognostic indicator across multiple cancer types treated with ICIs.^[11]^ Accordingly, patients were stratified into a high dNLR group (dNLR ≥ 3) and a low dNLR group (dNLR < 3).

#### 2.4.1. Efficacy assessment

Treatment efficacy was evaluated according to the response evaluation criteria in solid tumors.^[2]^ Treatment outcomes were categorized as complete response (CR), partial response (PR), stable disease (SD), and progressive disease (PD). The objective response rate (ORR) was defined as the percentage of patients achieving CR or PR, while the disease control rate (DCR) was defined as the percentage achieving CR, PR, or SD.

#### 2.4.2. Prognostic analysis

OS was defined as the time from initiation of ICI therapy to death or the date of last follow-up. PFS was defined as the time from the start of ICI therapy to disease progression or last follow-up.

### 2.5. Statistical analysis

Statistical analyses were conducted using SPSS software (version 27.0; IBM Corp., Armonk ) with precision. Quantitative data that met the criteria for normal distribution were assessed for intergroup differences using independent sample *t*-tests, with results reported as mean ± standard deviation. Categorical data were summarized as frequencies and percentages, and associations between these variables were evaluated using chi-square (χ^2^) tests. Missing data accounted for <5% of all variables. Continuous variables with missing values were imputed using the mean of available observations, while categorical variables were filled using the mode. Given the small proportion of missing data, this imputation method was considered appropriate and unlikely to affect the robustness of the results. For survival analysis, univariate assessments were performed utilizing Kaplan–Meier survival curves and log-rank tests, while multivariate analyses were executed using Cox proportional hazards regression models. Multivariate Cox regression models were adjusted for key clinical variables, including demographic factors (age, sex), functional status (Eastern Cooperative Oncology Group [ECOG] score), disease burden (tumor location, liver metastasis, number of metastatic sites, ascites, pleural effusion), treatment history (prior chemotherapy lines, smoking history), and treatment modality (ICI monotherapy, ICI plus chemotherapy, or ICI plus antiangiogenic therapy). To address potential concerns regarding the relatively small proportion of patients in the high dNLR group, a post hoc statistical power analysis was performed. Power calculations were based on the observed hazard ratios for PFS and OS between the high and low dNLR groups. Assuming a 2-sided α of 0.05, the sample size of 168 patients (38 in the high dNLR group and 130 in the low dNLR group) provided >80% power to detect the observed differences in survival outcomes. All statistical tests were 2-tailed, and a *P*-value of <.05 was considered indicative of statistical significance.

## 3. Results

### 3.1. Patient demographics and baseline clinical characteristics

In this study, 168 patients with AGC were analyzed, with demographic and baseline clinical characteristics summarized in Table 1. The median age was evenly distributed, with 50.6% of patients aged ≥58 years and 49.4% under 58 years. Males represented a significant majority of the cohort, accounting for 75.0%, while females comprised 25.0%. Smoking history was equally balanced, with 50.6% reporting a history of smoking. Most patients (89.3%) had an ECOG performance status of 0 to 1, indicating a generally preserved functional capacity, while 10.7% had a score of ≥2. Only a small proportion presented with pleural fluid (3.6%), whereas the remaining 96.4% had no pleural effusion. Additionally, 20.8% had ascites at baseline, with 79.2% showing no evidence of ascites.

**Table 1:** Patient demographics and baseline clinical characteristics.

Characteristic	Category	Data (n = 168)
Age	≥58 yr	85 (50.6%)
	<58 yr	83 (49.4%)
Sex	Female	42 (25.0%)
	Male	126 (75.0%)
Smoking history	Yes	85 (50.6%)
	No	83 (49.4%)
ECOG performance status	≥2	18 (10.7%)
	0–1	150 (89.3%)
Pleural fluid	Present	6 (3.6%)
	Absent	162 (96.4%)
Ascites	Present	35 (20.8%)
	Absent	133 (79.2%)
Tumor location	Cardia	29 (17.3%)
	Body/fundus	84 (50.0%)
	Pylorus	55 (32.7%)
Liver metastasis	Present	83 (49.4%)
	Absent	85 (50.6%)
Number of metastatic sites	0–2	122 (72.6%)
	≥3	46 (27.4%)
Prior chemotherapy lines	1	55 (32.7%)
	≥2	113 (67.3%)
Response to line before immunotherapy	PD	124 (73.8%)
	Others	44 (26.2%)
Dosage of immunotherapy	≥200 mg	150 (89.3%)
	<200 mg	18 (10.7%)
Combined with chemotherapy	Yes	115 (68.5%)
	No	53 (31.5%)
dNLR	<3	130 (77.4%)
	≥3	38 (22.6%)

dNLR = derived neutrophil-to-lymphocyte ratio, ECOG = Eastern Cooperative Oncology Group, PD = progressive disease.

Regarding tumor location, 50.0% of tumors were located in the body or fundus of the stomach, 32.7% in the pylorus, and 17.3% in the cardia. Liver metastasis was identified in 49.4% of patients, with the remaining 50.6% having no liver involvement. The majority of patients (72.6%) had 0 to 2 metastatic sites, while 27.4% presented with ≥3 metastatic sites. Prior chemotherapy was common, with 67.3% having received 2 or more prior lines of chemotherapy, and 32.7% undergoing only 1 line of chemotherapy before the initiation of immunotherapy. In terms of response to prior treatment, a substantial 73.8% had PD, with 26.2% categorized under other response types. Most patients (89.3%) received a high dosage of immunotherapy (≥200 mg), and 68.5% were treated with a combination of immunotherapy and chemotherapy. Finally, based on the dNLR, 77.4% of patients were in the low dNLR group (<3), while 22.6% had a dNLR of ≥3 (Table 1).

### 3.2. Comparison of treatment efficacy between dNLR < 3 and dNLR ≥ 3 groups

In multivariate Cox regression models adjusting for the clinical variables, elevated dNLR (≥3) remained independently associated with unfavorable outcomes. Specifically, patients with high dNLR had a significantly shorter PFS compared with those with low dNLR (adjusted hazard ratio [HR] = 1.872, 95% confidence interval [CI]: 1.184–2.961, *P* = .006). Similarly, OS was significantly reduced in the high dNLR group (adjusted HR = 1.801, 95% CI: 1.176–2.915, *P* = .008). Among other covariates, ECOG score ≥2 and ≥3 metastatic sites were also significantly correlated with poorer survival, while age, sex, and prior chemotherapy lines were not independently associated with outcomes. Treatment modality (ICI monotherapy vs combination therapy) did not alter the prognostic effect of dNLR. These findings reinforce that dNLR is an independent prognostic biomarker in AGC patients receiving ICIs, irrespective of other clinical characteristics and treatment strategies.

### 3.3. Comparison of treatment efficacy between dNLR < 3 and dNLR ≥ 3 groups

In evaluating the efficacy of immunotherapy across different dNLR levels, no statistically significant differences were observed between patients with dNLR < 3 and those with dNLR ≥ 3 (all *P* > .7). Among patients with low dNLR, the rates of CR and PR were 1.5% and 24.6%, respectively, compared with 0% and 23.7% in the high dNLR group (CR: OR = 0.94, 95% CI: 0.21–4.32, *P* = .998; PR: OR = 1.11, 95% CI: 0.42–2.77, *P* = .840). SD occurred in 23.1% versus 21.1% (OR = 0.85, 95% CI: 0.31–2.36, *P* = .761), while PD rates were 50.8% versus 55.2% (OR = 1.08, 95% CI: 0.47–2.52, *P* = .853) in the low and high dNLR groups, respectively. The overall DCR (CR + PR + SD) was slightly higher in the low dNLR group (49.2% vs 44.8%), but without statistical significance (OR = 0.92, 95% CI: 0.40–2.95, *P* = .853). Similarly, ORR (CR + PR) showed minimal variation between groups (26.2% vs 23.7%; OR = 1.04, 95% CI: 0.39–2.77, *P* = .932). These results confirm that, although patients with lower dNLR values exhibited marginally higher response rates, dNLR as a single biomarker did not significantly affect short-term treatment efficacy in this cohort (Table 2).

**Table 2:** Comparison of efficacy between dNLR < 3 and dNLR ≥ 3 groups (n, %).

Indicator	dNLR < 3 (n = 130)	dNLR ≥ 3 (n = 38)	*P*-value	OR	OR (95% CI)
CR	2 (1.5%)	0 (0.0%)	.998	0.94	0.21–4.32
PR	32 (24.6%)	9 (23.7%)	.840	1.11	0.42–2.77
SD	30 (23.1%)	8 (21.1%)	.761	0.85	0.31–2.36
PD	66 (50.8%)	21 (55.2%)	.853	1.08	0.47–2.52
DCR (CR + PR + SD)	64 (49.2%)	17 (44.8%)	.853	0.92	0.40–2.95
ORR (CR + PR)	34 (26.2%)	9 (23.7%)	.932	1.04	0.39–2.77

CI = confidence interval, CR = complete response, DCR = disease control rate, dNLR = derived neutrophil-to-lymphocyte ratio, OR = odds ratio, ORR = objective response rate, PD = progressive disease, PR = partial response, SD = stable disease.

### 3.4. Overall survival outcomes based on dNLR levels

In the cohort of 168 AGC patients, the median OS was 21.5 months, reflecting the baseline survival outcome for this population undergoing immunotherapy. OS varied notably between patients with differing baseline dNLR levels. Patients in the high dNLR group (dNLR ≥ 3) experienced a median OS of 15.6 months, significantly shorter than the median OS of 23.5 months observed in the low dNLR group (dNLR < 3). The HR for progression in the high dNLR group compared to the low dNLR group was 1.865, with a 95% CI of 1.216 to 3.156, indicating a nearly twofold increased risk of disease progression associated with elevated dNLR levels (*P* < .001). These findings underscore the prognostic value of dNLR as a biomarker, suggesting that patients with higher baseline dNLR may have poorer outcomes with respect to disease progression, potentially due to an unfavorable inflammatory and immunosuppressive tumor microenvironment.

### 3.5. Progression-free survival outcomes based on baseline dNLR levels

In this study cohort, the median PFS for all AGC patients undergoing immunotherapy was 12.8 months. When stratified by baseline dNLR, significant differences emerged between the high and low dNLR groups. Patients with high dNLR (≥3) demonstrated a notably shorter median PFS of 8.5 months, compared to a median PFS of 15.6 months in the low dNLR group (<3). This difference was statistically significant, with a HR of 1.916 (95% CI: 1.213–3.028, *P* < .001), indicating that patients with elevated dNLR had an almost twofold increase in the risk of disease progression compared to those with lower dNLR levels. These findings suggest that a high baseline dNLR is associated with a more aggressive disease course and decreased benefit from immunotherapy, potentially due to an inflammatory and immunosuppressive environment linked with poor tumor control.

### 3.6. Subgroup and multivariate analyses based on treatment regimens

To evaluate the potential confounding effect of treatment heterogeneity, exploratory subgroup analyses were performed according to treatment modality (ICI monotherapy vs ICI combined with chemotherapy vs ICI combined with antiangiogenic therapy). No significant interaction was observed between dNLR levels and treatment modality in relation to PFS or OS (all interaction *P* > .05). In addition, multivariate Cox regression models adjusting for treatment regimen (including ICI drug type and combination strategy) confirmed that elevated dNLR (≥3) remained an independent prognostic factor for both PFS (adjusted HR = 1.872; 95% CI: 1.184–2.961; *P* = .006) and OS (adjusted HR = 1.801; 95% CI: 1.176–2.915; *P* = .008). These findings suggest that the prognostic effect of baseline dNLR was not solely attributable to differences in treatment regimen.

### 3.7. Post hoc power analysis

To further evaluate the robustness of our findings given the smaller sample size in the high dNLR group, a post hoc power analysis was conducted. Using the observed hazard ratio for PFS (HR = 1.916; 95% CI: 1.213–3.028) and OS (HR = 1.865; 95% CI: 1.216–3.156), the calculated statistical power exceeded 80% at a significance level of .05. These results indicate that the study was sufficiently powered to detect significant differences in survival outcomes despite the limited number of patients in the high dNLR group. However, the analysis also confirmed that statistical power for secondary outcomes such as ORR and DCR was limited, which may partially explain the absence of significant differences between groups in these endpoints.

## 4. Discussion

The dNLR has garnered interest as a prognostic biomarker in oncology, especially within the context of ICI therapy. Elevated dNLR, reflecting a higher proportion of neutrophils relative to lymphocytes, is thought to represent a pro-inflammatory and immunosuppressive state that may hinder antitumor immunity.^[12,13]^ In patients with AGC, where survival outcomes remain poor despite advancements in treatment, identifying reliable biomarkers for predicting responses to ICIs is critical.^[14]^ ICIs, such as programmed death-1 and PD-L1 inhibitors, have shown promise in AGC, but patient responses are highly variable, underscoring the need for effective predictors of treatment efficacy.^[15–17]^ Research suggests that a high dNLR is associated with an unfavorable tumor microenvironment, characterized by reduced T-cell activation and increased tumor-promoting inflammation, which may impair the efficacy of ICIs. This study evaluated the dNLR as a predictive biomarker for prognosis in AGC patients undergoing ICI therapy. Our findings revealed that while there were no significant differences in the ORR and DCR between high and low dNLR groups, dNLR levels significantly impacted PFS and OS. Patients with a dNLR < 3 demonstrated a notably longer PFS and OS compared to those with dNLR ≥ 3, indicating that elevated dNLR levels are associated with a poorer prognosis in AGC.

One possible explanation for the association between elevated dNLR and poor prognosis lies in the role of systemic inflammation and immune suppression in cancer progression. An elevated dNLR, which indicates a higher ratio of neutrophils to lymphocytes, has been linked with increased levels of circulating neutrophils and reduced lymphocyte activity. Neutrophils contribute to an inflammatory tumor microenvironment by releasing cytokines, growth factors, and reactive oxygen species, all of which can promote tumor growth, angiogenesis, and metastasis. Additionally, neutrophils can suppress cytotoxic T-cell responses through various mechanisms, such as the secretion of arginase-1 and reactive oxygen species, thereby reducing the immune system’s capacity to effectively target and eliminate tumor cells.^[18,19]^ This inflammatory environment likely contributes to the limited efficacy of ICI therapy observed in patients with higher dNLR levels, as ICIs rely on robust T-cell activity to mediate their antitumor effects.

The significant disparity in PFS and OS between the dNLR < 3 and dNLR ≥ 3 groups further suggests that dNLR reflects more than just an inflammatory marker; it may also indicate the degree of immune suppression within the tumor microenvironment. Lymphocytes, particularly T cells, play a crucial role in the antitumor immune response. A lower lymphocyte count, as suggested by a high dNLR, may reflect a diminished pool of immune cells available to respond to ICIs. In the context of ICI therapy, which depends on reactivating T cells to recognize and kill cancer cells, a high dNLR may indicate a reduced likelihood of therapeutic success due to a compromised immune system.^[20,21]^ This could explain why, in our study, patients with low dNLR values experienced longer PFS and OS, as they may have a more intact immune system capable of mounting a stronger response to ICIs.

Although no significant differences were observed in ORR and DCR between the low and high dNLR groups, patients with elevated dNLR had significantly shorter PFS and OS. One possible explanation is that response evaluation criteria in solid tumors criteria, which focus on measurable tumor shrinkage, may not fully capture the immunotherapy-related dynamics such as pseudoprogression or delayed responses. Therefore, dNLR may reflect the broader immune-inflammatory status influencing long-term tumor control rather than short-term radiographic changes. Another potential factor is the effect of subsequent treatments after disease progression. In this cohort, most patients received second-line or later therapies following progression, including chemotherapy, antiangiogenic therapy, or best supportive care. However, treatment strategies varied substantially, and detailed data were incomplete in this retrospective analysis, which limited our ability to fully evaluate their impact on long-term outcomes. It is plausible that differences in post-progression management may have contributed to variations in OS, although the consistency between PFS and OS findings suggests that baseline dNLR exerts an independent prognostic effect. These observations indicate that dNLR may primarily serve as a prognostic rather than a predictive biomarker, reflecting systemic immune competence that influences overall disease trajectory beyond initial radiographic response. Future prospective studies with comprehensive recording of post-progression therapies and the use of immune-related response criteria may help further clarify the mechanisms underlying these discrepancies.

Moreover, the failure of dNLR to significantly impact ORR and DCR could be attributed to the complex interaction between inflammation and immune evasion in AGC. While dNLR can reflect systemic inflammation, it does not fully capture other tumor-intrinsic factors, such as PD-L1 expression, microsatellite instability, or tumor mutational burden, which are known to influence the efficacy of immunotherapy.^[22,23]^ This complexity suggests that while dNLR provides a snapshot of systemic inflammation, it may be insufficient as a standalone marker for predicting response rates to ICIs. However, its significant association with PFS and OS highlights its value as a prognostic biomarker, particularly in the context of long-term outcomes rather than immediate treatment response.^[24,25]^ Our findings align with previous research that identifies elevated dNLR as a poor prognostic marker in various cancers treated with ICIs, including lung and renal cancers. The consistency of these findings across different cancer types highlights the role of systemic inflammation in limiting the benefits of ICIs and supports the use of dNLR as a broadly applicable prognostic indicator. Nevertheless, it is essential to acknowledge that dNLR alone may not suffice as a predictive biomarker, and integrating it with other markers, such as PD-L1 expression or specific genetic alterations, could enhance its prognostic accuracy. The clinical implications of these results suggest that baseline dNLR could be used as a stratification tool for AGC patients undergoing ICI therapy. Patients with a high dNLR might theoretically benefit from future therapeutic approaches aimed at modulating the inflammatory microenvironment.^[26,27]^ For instance, combining ICIs with anti-inflammatory agents or therapies targeting neutrophil recruitment could be explored in preclinical or early clinical studies; however, this remains a hypothesis that requires further experimental and clinical validation. Additionally, patients with high dNLR may warrant closer monitoring and more proactive management due to their increased risk of disease progression, as indicated by shorter PFS and OS.

In addition to dNLR, several hematologic inflammatory biomarkers, including the NLR and the platelet-to-lymphocyte ratio, have been investigated as prognostic indicators in various malignancies. NLR reflects the balance between systemic inflammation and adaptive immune response, while platelet-to-lymphocyte ratio is thought to capture the interplay between platelet-driven tumor angiogenesis and lymphocyte-mediated immune surveillance. Although both markers have shown prognostic relevance in AGC, their values may be influenced by dynamic changes in total leukocyte or platelet counts, which can be affected by concurrent conditions such as infection, thrombocytosis, or treatment-related toxicities. In contrast, dNLR incorporates neutrophils in relation to the combined pool of lymphocytes and monocytes, thereby offering a simplified yet stable parameter that can be readily derived from standard complete blood count data. Importantly, previous studies have suggested that dNLR may demonstrate prognostic performance comparable to or superior to NLR across multiple cancer types, while requiring no additional laboratory testing. Taken together, these characteristics highlight the clinical utility of dNLR as a practical, cost-effective, and reproducible biomarker for risk stratification in patients receiving ICIs.

This study has several limitations that should be acknowledged. First, its retrospective and single-center design may introduce inherent selection bias and limit the generalizability of the findings. Potential sources of selection bias include the relatively long enrollment period (2015–2019), during which clinical practice patterns and accessibility of ICIs evolved, as well as the varying eligibility criteria and follow-up durations among patients. Second, treatment heterogeneity also existed, as patients received different ICIs and combination regimens (monotherapy, chemo-immunotherapy, or ICI with antiangiogenic therapy). Although subgroup analyses suggested that the prognostic value of dNLR was consistent across treatment modalities, residual confounding cannot be completely excluded. Third, the relatively small sample size of the high dNLR group (n = 38) may have reduced the statistical power for secondary endpoints such as ORR and DCR, even though post hoc power analysis confirmed adequate power (>80%) for survival outcomes. Fourth, the cutoff value of dNLR was set at 3 based on previously published international studies; while this facilitates comparison with existing literature, its optimality for AGC was not validated in this cohort. Finally, information on post-progression treatments was not uniformly available, which may have influenced OS outcomes. Future prospective, multicenter studies with larger cohorts and standardized treatment strategies are needed to validate these findings and to explore integrated biomarker models that combine dNLR with other clinical and molecular predictors.

## 5. Conclusions

In conclusion, pretreatment dNLR levels serve as an independent prognostic biomarker for PFS and OS in AGC patients undergoing immunotherapy. Patients with low baseline dNLR levels may derive greater benefit from ICI therapy, highlighting the potential of dNLR in guiding treatment decisions and optimizing patient outcomes.

## Acknowledgments

We appreciate the cooperation and informed consent provided by the patients for this study.

## Author contributions

**Conceptualization:** Yu Cao, Hangjun Gong, Yun Zhang, Xu Zhang.

**Data curation:** Yu Cao, Hangjun Gong, Yanyan Zhang.

**Formal analysis:** Yu Cao, Hangjun Gong, Gang Han, Yun Zhang.

**Funding acquisition:** Yu Cao, Gang Han, Yun Zhang.

**Investigation:** Yu Cao, Gang Han.

**Writing – original draft:** Yu Cao, Gang Han, Yun Zhang, Yanyan Zhang, Xu Zhang.

**Writing – review & editing:** Yu Cao, Gang Han, Yun Zhang, Yanyan Zhang, Xu Zhang.

## References

[1] Guan WL He Y Xu RH. Gastric cancer treatment: recent progress and future perspectives. J Hematol Oncol. 2023;16:57. 37245017 10.1186/s13045-023-01451-3 PMC10225110

[2] Gouda MA Janku F Wahida A. Liquid biopsy response evaluation criteria in solid tumors (LB-RECIST). Ann Oncol. 2024;35:267–75. 38145866 10.1016/j.annonc.2023.12.007

[3] Matsuoka T Yashiro M. Novel biomarkers for early detection of gastric cancer. World J Gastroenterol. 2023;29:2515–33. 37213407 10.3748/wjg.v29.i17.2515 PMC10198055

[4] Li S Yu W Xie F. Neoadjuvant therapy with immune checkpoint blockade, antiangiogenesis, and chemotherapy for locally advanced gastric cancer. Nat Commun. 2023;14:8. 36596787 10.1038/s41467-022-35431-x PMC9810618

[5] Cheng R Li B Wang H Zeng Y. Immune checkpoint inhibitors and cellular immunotherapy for advanced gastric, gastroesophageal cancer: a long pathway. Clin Transl Oncol. 2023;25:3122–38. 37036597 10.1007/s12094-023-03181-x

[6] Zeng D Wu J Luo H. Tumor microenvironment evaluation promotes precise checkpoint immunotherapy of advanced gastric cancer. J ImmunoTher Cancer. 2021;9:e002467. 34376552 10.1136/jitc-2021-002467 PMC8356190

[7] Zeng Z Yang B Liao Z. Progress and prospects of immune checkpoint inhibitors in advanced gastric cancer. Future Oncol. 2021;17:1553–69. 33397136 10.2217/fon-2020-0829

[8] Nakazawa N Sohda M Tateno K. Albumin-derived neutrophil-to-lymphocyte ratio score as a marker of nivolumab treatment sensitivity in gastric cancer: a multicenter study. In Vivo. 2023;37:818–24. 36881071 10.21873/invivo.13147 PMC10026635

[9] Wan M Ding Y Mao C. Association of inflammatory markers with survival in patients with advanced gastric cancer treated with immune checkpoint inhibitors combined with chemotherapy as first line treatment. Front Oncol. 2022;12:1029960. 36387183 10.3389/fonc.2022.1029960 PMC9650180

[10] von Elm E Altman DG Egger M Pocock SJ Gøtzsche PC Vandenbroucke JP; STROBE Initiative. The Strengthening the Reporting of Observational Studies in Epidemiology (STROBE) statement: guidelines for reporting observational studies. J Clin Epidemiol. 2008;61:344–9. 18313558 10.1016/j.jclinepi.2007.11.008

[11] Mezquita L Auclin E Ferrara R. Association of the lung immune prognostic index with immune checkpoint inhibitor outcomes in patients with advanced non-small cell lung cancer. JAMA Oncol. 2018;4:351–7. 29327044 10.1001/jamaoncol.2017.4771 PMC5885829

[12] Akkanapally V Bai XF Basu S. Therapeutic immunomodulation in gastric cancer. Cancers (Basel). 2024;16:560. 38339311 10.3390/cancers16030560 PMC10854796

[13] Kim H Kim H Lee M. When to apply immune checkpoint inhibitor in patients with refractory advanced gastric cancer. J Cancer. 2021;12:5681–6. 34405028 10.7150/jca.62853 PMC8364657

[14] Ghidini M Petrillo A Botticelli A. How to best exploit immunotherapeutics in advanced gastric cancer: between biomarkers and novel cell-based approaches. J Clin Med. 2021;10:1412. 33915839 10.3390/jcm10071412 PMC8037391

[15] Li K Zhang A Li X Zhang H Zhao L. Advances in clinical immunotherapy for gastric cancer. Biochim Biophys Acta Rev Cancer. 2021;1876:188615. 34403771 10.1016/j.bbcan.2021.188615

[16] Zeng Z Zhu Q. Progress and prospects of biomarker-based targeted therapy and immune checkpoint inhibitors in advanced gastric cancer. Front Oncol. 2024;14:1382183. 38947886 10.3389/fonc.2024.1382183 PMC11211377

[17] Wu YX Zhou X-Y Wang J-Q. Application of immune checkpoint inhibitors in immunotherapy for gastric cancer. Immunotherapy. 2023;15:101–15. 36597704 10.2217/imt-2022-0080

[18] Song S Li C Li S Gao H Lan X Xue Y. Derived neutrophil to lymphocyte ratio and monocyte to lymphocyte ratio may be better biomarkers for predicting overall survival of patients with advanced gastric cancer. Onco Targets Ther. 2017;10:3145–54. 28706446 10.2147/OTT.S138039 PMC5495088

[19] El Helali A Tao J Wong CHL. A meta-analysis with systematic review: efficacy and safety of immune checkpoint inhibitors in patients with advanced gastric cancer. Front Oncol. 2022;12:908026. 36387109 10.3389/fonc.2022.908026 PMC9660259

[20] Matsas S Aguiar PN Jr Del Giglio A. Neutrophil-to-lymphocyte ratio and platelet-to-lymphocyte ratio as biomarkers to prognosticate survival in advanced gastric cancer patients in the era of immunotherapy: a systematic review and meta-analysis. J Gastrointest Oncol. 2024;15:33–51. 38482212 10.21037/jgo-23-808 PMC10932683

[21] Rodrigues S Figueiredo C. Recent insights into the use of immune checkpoint inhibitors in gastric cancer. Porto Biomed J. 2022;7:e162. 35146175 10.1097/j.pbj.0000000000000162 PMC8824404

[22] Yoneda A Kuroki T Eguchi S. Immunotherapeutic advances in gastric cancer. Surg Today. 2021;51:1727–35. 33590326 10.1007/s00595-021-02236-2

[23] Togasaki K Sukawa Y Kanai T Takaishi H. Clinical efficacy of immune checkpoint inhibitors in the treatment of unresectable advanced or recurrent gastric cancer: an evidence-based review of therapies. Onco Targets Ther. 2018;11:8239–50. 30538493 10.2147/OTT.S152514 PMC6254591

[24] Han QY Zhang X Zhang J-G. Pre-operative neutrophil-to-lymphocyte ratio is an independent prognostic factor in patients with gastric cancer. Int Immunopharmacol. 2022;113:109371. 36279674 10.1016/j.intimp.2022.109371

[25] Chang X Ge X Zhang Y Xue X. The current management and biomarkers of immunotherapy in advanced gastric cancer. Medicine (Baltim). 2022;101:e29304. 10.1097/MD.0000000000029304 PMC9276259 35623069

[26] Abdel-Rahman O. Immune checkpoints aberrations and gastric cancer; assessment of prognostic value and evaluation of therapeutic potentials. Crit Rev Oncol Hematol. 2016;97:65–71. 26321371 10.1016/j.critrevonc.2015.08.015

[27] Lee CK Lee JB Park SJ. Second-line chemoimmunotherapy with nivolumab and paclitaxel in immune-related biomarker-enriched advanced gastric cancer: a multicenter phase Ib/II study. Gastric Cancer. 2024;27:118–30. 37906316 10.1007/s10120-023-01435-9

